# A comparison of sample preparation strategies for biological tissues and subsequent trace element analysis using LA-ICP-MS

**DOI:** 10.1007/s00216-016-0124-6

**Published:** 2016-12-14

**Authors:** Maximilian Bonta, Szilvia Török, Balazs Hegedus, Balazs Döme, Andreas Limbeck

**Affiliations:** 1 Institute of Chemical Technologies and Analytics, TU Wien, Getreidemarkt 9/164-IAC, 1060 Vienna, Austria; 2 Department of Tumor Biology, National Koranyi Institute of Pulmonology, Budapest, 1121 Hungary; 3 Division of Thoracic Surgery, Department of Surgery, Comprehensive Cancer Center Vienna, Medical University of Vienna, 1090 Vienna, Austria

**Keywords:** Biological samples, Laser ablation, Mass spectrometry ICP-MS

## Abstract

Laser ablation-inductively coupled plasma-mass spectrometry (LA-ICP-MS) is one of the most commonly applied methods for lateral trace element distribution analysis in medical studies. Many improvements of the technique regarding quantification and achievable lateral resolution have been achieved in the last years. Nevertheless, sample preparation is also of major importance and the optimal sample preparation strategy still has not been defined. While conventional histology knows a number of sample pre-treatment strategies, little is known about the effect of these approaches on the lateral distributions of elements and/or their quantities in tissues. The technique of formalin fixation and paraffin embedding (FFPE) has emerged as the gold standard in tissue preparation. However, the potential use for elemental distribution studies is questionable due to a large number of sample preparation steps. In this work, LA-ICP-MS was used to examine the applicability of the FFPE sample preparation approach for elemental distribution studies. Qualitative elemental distributions as well as quantitative concentrations in cryo-cut tissues as well as FFPE samples were compared. Results showed that some metals (especially Na and K) are severely affected by the FFPE process, whereas others (e.g., Mn, Ni) are less influenced. Based on these results, a general recommendation can be given: FFPE samples are completely unsuitable for the analysis of alkaline metals. When analyzing transition metals, FFPE samples can give comparable results to snap-frozen tissues.

**Graphical abstract:**
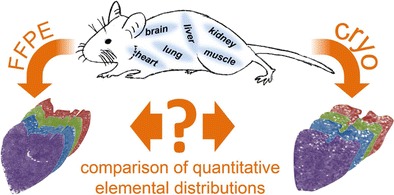
Sample preparation strategies for biological tissues are compared with regard to the elemental distributions and average trace element concentrations.

Sample preparation strategies for biological tissues are compared with regard to the elemental distributions and average trace element concentrations.

## Introduction

Laser ablation-inductively coupled plasma-mass spectrometry (LA-ICP-MS) is a tool nowadays widely used for the laterally resolved assessment of trace elements in biological tissues [1]. Exceptional limits of detection for a wide range of metallic analytes and good lateral resolutions combined with reasonable acquisition times make this technique a valuable tool for such imaging applications [2, 3]. During the last decades, a variety of works on bio-imaging using LA-ICP-MS have been presented and the technique has already been included in clinical research in some examples. The tissue type investigations have been performed on (e.g., liver [4], brain [5, 6], kidney [7], or tumor tissues [8, 9]) are as numerous as the elements that have been analyzed already; here, naturally occurring minor (e.g., Na, K) and trace elements (e.g., Ni, Cu, Zn), as well as elements artificially introduced during chemotherapy (e.g., Pt [8, 9]) or being used as contrasting agents (e.g., Gd [10]), have to be named in this context. LA-ICP-MS does not only give the opportunity of acquiring qualitative distribution images but also quantification is possible [11, 12]. With regard to the quantification strategy, a wide variety of approaches has been presented in the past, as LA-ICP-MS suffers from severe matrix effects and slight variations in tissue composition may already induce unwanted signal changes that do not reflect the actual composition of the sample. Thus, the use of appropriate standard materials, as well as employing a suitable internal standard, is imperative [13]. As certified reference materials are scarcely available for biological tissues, alternative approaches have to be used ranging from the preparation of gel standards [14], over printed patterns on paper [9], to the use of polymeric layers [15], or the online addition of aqueous standards [16]. Also concerning internal standardization, possibilities are various, including not only the sample inherent carbon but also additionally applied materials/reagents such as thin gold layers [17, 18], protein-metal tags [19], or polymer thin films [15]. Each of the named approaches has their advantages and weaknesses. Their description is, however, not within the scope of this work.

However, an important part of accurate analysis is sample preparation and pretreatment. Here, some research is required to evaluate appropriate strategies for obtaining reliable results from bio-imaging experiments. Typical histology knows a variety of sample preparation techniques, each one fit for different purposes, whether performing conventional histological staining, immunohistochemistry, or other, more specialized, techniques. Despite the variety of sample fixation, preparation, and cutting strategies, one method has emerged as the gold standard: formalin fixation and paraffin embedding (FFPE) [20]. Main advantages over other techniques are that the samples can be stored under ambient conditions for decades and thin cutting is fairly simple and can also be carried out at room temperature, i.e., no cryo-microtome is required. Due to the exceptional storage properties, large tissue archives of FFPE tissues are available, even from very rare diseases. However, the sample preparation process involves a number steps which might alter the actual elemental distribution. During formalin fixation, the tissue specimen is immersed in a solution of formaldehyde in water (known as formalin), resulting in cross-linking of proteins within the tissue. Subsequently, water in the tissue is substituted by an organic solvent (often xylene) in several solvent exchange steps. Finally, the sample is embedded in paraffin; this sample block can further be used to prepare thin cuts of the sample. Thin cuts are deposited on suitable sample carrier material (e.g., microscopic slides, silicon wafers) before the paraffin is removed. This involves an additional washing step using an organic solvent. In contrast, elemental distributions and quantities in snap-frozen and unfixed tissue sections were expected to represent the actual in vivo conditions in the best way. This sample preparation strategy involves the least sample processing of all techniques; tissue specimen are snap frozen in liquid nitrogen and thin cuts of some micrometer thickness are prepared in a cryotome without a fixation step. Major disadvantage is that unfixed tissues are scarcely available for clinical studies mainly due to reasons of storage and handling. Cryo-samples need to be stored at −80 °C to avoid proteolytic reactions, which is an important fact hampering general applicability of this sample preparation approach. The suitability of FFPE tissues for molecular analysis techniques has already been investigated earlier [21]. Also, bulk element concentrations of selected metals in specific tissues have already been compared between FFPE sample preparation and cryo-sectioning [22, 23]. However, to the best of the authors’ knowledge, no study including multiple organs and physiologically relevant metals ranging from alkalines over earth alkalines to transition metals has been presented where elemental distributions as well as bulk concentrations have been compared.

In this work, the suitability of FFPE sample preparation for trace elemental distribution analysis using LA-ICP-MS is examined. Organs from one single mouse were partly used for FFPE sample preparation and for cryo-preparation, which is considered being the reference method. By using the same organ of one mouse for both preparation techniques, possible biological variations are sought to be reduced to a minimum. While the tissue has to undergo several washing steps where ionic species may potentially be washed out or their spatial distribution may be altered, the preparation for cryo-cutting is far more straightforward, leading to the probably most accurate picture of the physiological conditions. Elemental concentrations of nine elements (Na, Mg, K, Ca, Mn, Fe, Ni, Cu, and Zn) as well as their distributions were examined and compared leading to a comprehensive picture of the suitability of FFPE sample preparation for elemental imaging experiments.

## Experimental

### Chemicals

Xylene (isomer mixture, histological grade), aqueous formaldehyde solution (≥36.0%, for molecular histology), and paraffin wax (for histology) were all purchased from Sigma Aldrich, Buchs, Switzerland. Ultra-pure water (resistivity 18.2 MΩ cm) was dispensed from a Barnstead EASYPURE II water system (ThermoFisher Scientific, Marietta, OH). Ethanol, toluene, (3-aminopropyl)-triethoxysilane (APES), acetone, and nitrate salts of Na, K, Mg, Mn, Fe, Cu, and Zn were all of p.a. quality and purchased from Sigma Aldrich, Buchs, Switzerland. Conc. HNO_3_ was of p.a. quality and obtained from Merck, Darmstadt, Germany.

### Instrumental

All LA-ICP-MS measurements were performed using an NWR213 LA system (ESI, Fremont, CA) equipped with fast washout cell and a 213-nm frequency quintupled Nd:YAG laser coupled to an iCAP Qc ICP-MS instrument (ThermoFisher Scientific, Bremen, Germany). Coupling of the two instruments was established using a 1.0-m-long PTFE tubing with an inner diameter of 1.0 mm. Laser ablation parameters were optimized in preliminary experiments to ablate the complete sample material in one run of analysis while maintaining the integrity of the sample material surrounding the area of the incident laser beam. A lateral resolution of 40 μm was selected to be appropriate for the performed investigations; this value provided a good trade-off between time-efficient measurement and good image quality. While all instrumental settings for the LA device were kept constant throughout all measurements, the ICP-MS had to be tuned on a daily basis to obtain best sensitivity for the measurements. Optimization was performed using the very same dried droplet standards used for signal quantification. A dried droplet standard prepared using the highest analyte concentration (10.0 mg L^−1^ Na, K, and Mg; 4.0 mg L^−1^ Ca and Fe; 2.0 mg L^−1^ Zn; 1.0 mg L^−1^ Mn; and 0.40 mg L^−1^ Ni and Cu) was used. Performance was optimized to reach maximum signal intensities for ^23^Na, ^56^Fe, ^63^Cu, and ^64^Zn; typical measurement parameters are summarized in Table 1.

**Table 1:** Summary of instrumental parameters used for the LA-ICP-MS imaging experiments

Laser ablation		ICP-MS	
Wavelength	213 nm	Plasma power	1550 W
Pulse duration	4 ns	Cool gas flow	14.0 L min^−1^
Laser repetition rate	20 Hz	Auxiliary flow	0.8 L min^−1^
Laser beam diameter	40 μm	Cones	Ni
Laser energy	3.19 mJ	Scanning mode	Peak hopping
Laser scan speed	120 μm s^−1^	Dwell time per isotope	10 ms
Laser beam geometry	Circular	Monitored isotopes	^13^C, ^23^Na, ^24^Mg, ^25^Mg, ^39^K, ^42^Ca, ^44^Ca, ^55^Mn, ^56^Fe, ^57^Fe, ^58^Ni, ^60^Ni, ^63^Cu, ^64^Zn, ^65^Cu, ^66^Zn, ^197^Au
He gas flow	1.0 L min^−1^	Mass resolution at *m*/*z* 238	300 m/Δm
Ar make-up flow	0.8 L min^−1^		

Gold coating of standards and samples was performed using an Agar B7340 sputter coater (Agar Scientific Limited, Essex, UK) equipped with a gold target. Sputter parameters were kept constant over all experiments, as reported in a previous study [18]. Microscopic images of the samples were acquired using a light microscope operated in reflective light mode at 50× magnification (Leica DM2500M, Leica Microsystems, Wetzlar, Germany).

### Sample preparation and ICP-MS measurement

The animal-model protocol was developed and conducted in accordance with the ARRIVE guidelines and the animal welfare regulations of the Department of Tumor Biology, National Koranyi Institute of Pulmonology (permission number: 22.1/1268/3/2010). Mice were kept on a daily 12-h light/12-h dark cycle and held in conventional animal house in microisolator cages with water and laboratory chow ad libitum. After sacrification, five organs (heart, lung, kidney, liver, and brain) as well as pieces of muscle tissue were removed from the animals and each divided into two halves. One half underwent the complete FFPE procedure, whereas the other half was snap frozen in liquid nitrogen directly after sampling and stored at −80 °C until further use. This procedure was found appropriate to obtain optimal comparability between FFPE and cryo-cut samples. During the sample preparation process and sample cutting, care was taken to keep both samples in a similar orientation, which would then facilitate inter-sample comparison. Even though this procedure showed to be more complicated than sampling the organs from two individual mice, it offered the opportunity to exclude the influence of biological variation between two specimens.

In the preparation of the cryo-cuts, the tissue specimen was attached to a sample holder using the Shandon Cryomatrix (Thermo Scientific, Cat. No: 6769006). At −20 °C, cryo-cuts with 10 μm thickness were prepared using a cryotome (Leica CM3050 S, Leica Microsystems, Wetzlar, Germany). Slices were deposited onto squared 1 × 1 cm surface-modified silicon wafers for minimization of background signals originating from the carrier material—a necessity for high sensitivity trace element measurements with optimal quantification accuracy. Silicon wafers (Infineon Technologies Austria AG, Villach, Austria) were surface coated before deposition of the tissue samples to ensure optimal adhesion of the thin sections. The surface coating was performed as reported previously [24]. Samples were allowed to dry at room temperature before further analysis. FFPE samples were prepared according to standard procedures used in clinical laboratories. In analogy to the cryo-cuts, they were also cut to 10 μm thickness and deposited on silicon wafers; the thin cuts were washed with toluene to remove excess paraffin.

Before LA-ICP-MS analysis, all samples were coated with a thin gold layer to be utilized as a pseudo-internal standard. Previous experiments showed that deposition of the gold layer is very reproducible with relative standard deviations of the amount of deposited material below 4%. This metal layer with a thickness in the nanometer range is ablated simultaneously with the sample material. It has been shown in earlier studies that such signal normalization approach allows the compensation of matrix effects (i.e., material ablation and transport), instrumental drifts during measurement time, and day-to-day signal variations [18].

For quantification of trace elements in the tissue samples, a dried droplet approach reported earlier to deliver valid results for tissue investigations [25] has been employed. In this simple method, defined amounts of liquid standards are deposited onto pre-cut filter pieces and allowed to dry. A stock solution containing 10.0 mg L^−1^ Na, K, and Mg; 4.0 mg L^−1^ Ca and Fe; 2.0 mg L^−1^ Zn; 1.0 mg L^−1^ Mn; and 0.40 mg L^−1^ Ni and Cu in 1% nitric acid (*v*/*v*) was used for preparation of the dried droplet standards. A standard series was generated by dilution of this stock by factors 2.0, 3.0, 5.0, 10.0, and 20.0. The stock itself and a blank solution without analyte addition were also used for preparation of the dried droplets. Every liquid standard, a concentration of 1.0 mg L^−1^ indium was added to serve as internal standard for correction of inaccuracies during pipetting. Ten microliters of standard solution were applied to each filter piece, six replicates of each concentration level were prepared. For the standards, gold normalization is applied in the same manner as for the samples: After evaporation of the liquid, a pattern of dried droplets is coated with a thin layer of gold. The same instrumental parameters as for the tissue samples are used, ensuring comparable thickness of the gold layers on samples and standards. Measurement of the standards was performed using the very same laser parameters than for the images; just the stage scan speed has been reduced to 40 μm s^−1^ in order to ablate the complete filter material. Using radial line scans, one line across the diameter of the filter was ablated, derived signals were normalized and averaged, and a calibration function to be used for signal quantification was calculated. Using this calibration function, area concentrations of each analyte in the tissue can be calculated (e.g., ng cm^−2^) which can then be further transformed into mass concentrations knowing the tissue thickness and the density of the wet tissue [25].

## Results and discussion

With six tissue types, each measured in three replicates, and two different sample preparation strategies, a total of 36 tissue samples were investigated in this comparative study between FFPE and snap-frozen tissue preparation. Dimensions of the tissues ranged from approx. 3 × 3 to 8 × 8 mm^2^. LA-ICP-MS measurements provided signals above background level for all selected elements on all samples. Normalized signal intensities for each pixel were within the signal range obtained for the dried droplet calibrations. Quantification of the obtained signal intensities was performed by transforming the data matrix of each element using the calibration functions from the dried droplet calibration. Derived element concentrations ranged between 0.1 μg g^−1^ (Mn) to 3000 μg g^−1^ (K). Whenever possible, two isotopes of each element were monitored and quantification was performed. The isotope with the higher natural abundance was always used for data evaluation, the other one for quality control purposes. In all cases, the distribution images of two isotopes were well correlated; calculated analyte concentrations did not differ significantly. Repeatability of the measurements was validated using consecutive tissue slices.

**Fig. 1:**
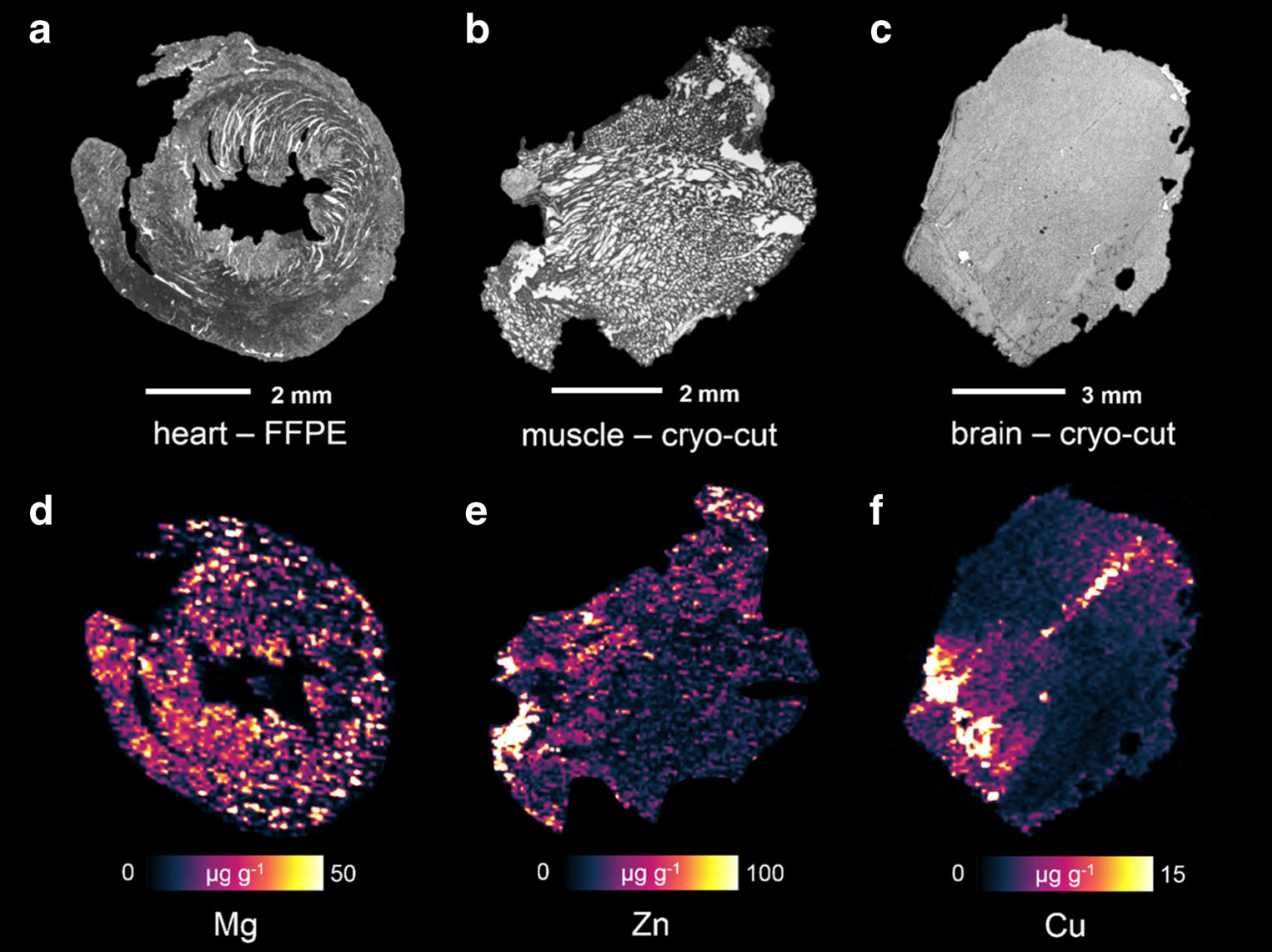
Elemental distributions of Mg (**d**) in a FFPE-treated heart section cut along the transverse plane, Zn distribution in a cryo-cut muscle sample (**e**), as well as Cu distribution in a cryo-cut coronal brain sample (**f**) with the corresponding micrographs of the heart (**a**), muscle (**b**), and brain (**c**)

Exemplary elemental distribution images from the LA-ICP-MS analyses are shown in Fig. 1. The distributions of Mg in heart tissue (FFPE; Fig. 1a, d), Zn in muscle tissue (cryo-cut; Fig. 1b, e), and Cu in brain tissue (cryo-cut; Fig. 1c, f) are shown.

Figure 1 demonstrates that the elemental distributions in the samples are inhomogeneous and vary with morphological structures of the tissue. Such findings were also reported earlier in other laterally resolved elemental distribution studies [26, 27]. Found distributions are in good accordance with previously reported data. Given that the analyzed sections are representative for the complete tissue/organ, these average concentrations across the complete section would correspond well with bulk metal concentrations. For calculation of average concentrations over the tissue material, the ^13^C signal was used as indicator for the presence of tissue material; a region of interest was defined for each tissue thin cut including all pixels where ^13^C signal was above background level. Additionally, visual interpretation was used to verify that the ^13^C signal did not originate from possibly remaining embedding medium. For each element, average concentrations over these regions of interest were calculated to yield an average concentration. Thereby calculated concentrations from the measurement of three consecutive tissue thin cuts were averaged to obtain a mean concentration and a standard deviation, respectively. The calculated bulk metal concentrations for all elements and all tissue types are shown in Table 2.

**Table 2:** Average concentrations of all measured elements in all analyzed tissues with a comparison between cryo-samples and FFPE preparation; all units are given in μg g^−1^, and errors are given as a single standard deviation (*n* = 3)

		Brain	Liver	Heart	Lung	Kidney	Muscle
Na	Cryo	528 ± 78.6	238 ± 2.83	347 ± 42.3	322.9 ± 33.3	1040 ± 91.3	610 ± 13.3
	FFPE	13.4 ± 1.82	9.32 ± 1.10	7.62 ± 1.16	2.2 ± 0.2	14.7 ± 2.19	7.53 ± 0.35
Mg	Cryo	78.9 ± 11.7	79.0 ± 2.57	61.0 ± 6.15	39.1 ± 5.0	116 ± 7.74	42.6 ± 6.82
	FFPE	44.9 ± 2.56	28.6 ± 1.94	25.8 ± 3.63	8.38 ± 0.6	63.0 ± 8.87	35.3 ± 4.97
K	Cryo	2510 ± 353	1470 ± 11.3	1230 ± 51	591 ± 78.9	2240 ± 215	743 ± 48.4
	FFPE	5.51 ± 0.55	5.27 ± 0.62	5.2 ± 0.8	2.14 ± 0.21	6.47 ± 0.45	5.17 ± 0.83
Ca	Cryo	73.8 ± 10.4	48.9 ± 4.23	97.9 ± 8.2	73.9 ± 10.3	80.2 ± 9.43	52.3 ± 3.23
	FFPE	822 ± 58.8	398 ± 55.8	393.2 ± 14.2	133 ± 17.7	647 ± 29.0	683 ± 81.0
Mn	Cryo	0.48 ± 0.05	2.37 ± 0.33	1.3 ± 0.1	0.12 ± 0.00	2.02 ± 0.27	0.25 ± 0.01
	FFPE	0.41 ± 0.05	2.26 ± 0.24	1.5 ± 0.1	0.12 ± 0.01	2.45 ± 0.12	0.31 ± 0.05
Fe	Cryo	29.0 ± 4.59	256 ± 29.3	167.0 ± 11.6	59.2 ± 3.27	140 ± 9.87	18.6 ± 1.29
	FFPE	24.3 ± 3.25	202 ± 25.4	143.4 ± 5.8	43.8 ± 0.95	106 ± 1.23	16.2 ± 2.26
Ni	Cryo	1.32 ± 0.11	1.92 ± 0.31	1.3 ± 0.1	0.55 ± 0.04	1.32 ± 0.24	0.88 ± 0.12
	FFPE	1.02 ± 0.10	1.78 ± 0.17	1.2 ± 0.1	0.45 ± 0.06	1.17 ± 0.12	0.95 ± 0.16
Cu	Cryo	5.29 ± 0.54	110 ± 7.13	13.1 ± 1.1	0.90 ± 0.08	4.32 ± 0.38	1.43 ± 0.13
	FFPE	6.27 ± 1.32	100 ± 10.6	11.5 ± 1.4	1.22 ± 0.24	5.04 ± 0.78	1.68 ± 0.21
Zn	Cryo	16.5 ± 1.44	23.7 ± 1.85	10.3 ± 1.4	9.96 ± 0.51	11.6 ± 1.77	23.9 ± 2.28
	FFPE	191 ± 22.6	90.4 ± 7.53	186.2 ± 12.2	19.3 ± 3.07	82.7 ± 6.63	178 ± 9.45

The average concentrations calculated from the elemental distribution images vary between 1.0 μg g^−1^ for nickel in brain tissue and 2500 μg g^−1^ for potassium also in brain tissue. The relative standard deviation of the calculated averages (*n* = 3) was usually below 15%, a value which is acceptable with regard to possible biological variations even within the same organ. To the best of the authors’ knowledge, this is the first study reporting average data as well as laterally resolved trace metal images from multiple elements in a large range of organs from one individual. Such extensive data set allows convenient investigations on the inter-relations of different elements in various tissue types. Found concentrations for Na and K are usually very high (lowest values of 238 μg g^−1^ for Na and 591 μg g^−1^ for K) in the analyzed tissues. This is explained by the fact that the alkaline elements are responsible for maintaining the physiological conditions and the osmotic pressure in cellular systems by creating ionic potentials [28]. The absolute concentrations as well as the ratios between the element concentrations of Na and K change with the respective tissue type but the K concentration is in all cases higher than the Na concentrations. Less variation in the absolute amounts and overall lower concentrations can be detected for the earth alkaline elements Mg and Ca. High concentrations and overall presence of these four elements can be explained by their general and wide purpose. As already mentioned, Na and K are part of every tissue to keep up physiologically suitable conditions. Besides also occurring in free form, Mg and Ca are also important cofactors in protein complexes, for example, in enzymes used for DNA replication, which takes place in every tissue type. All other elements (transition metals) are related to rather specific functions as cofactors in proteins and other macromolecules. Thus, their concentrations in different tissue types can change widely. As an example, the iron concentration is higher in tissues where blood is processed: liver, kidney, and heart. While the highest found concentration is in the liver (256 μg g^−1^), the lowest calculated average concentration is more than a factor of 10 below (18.6 μg g^−1^ in the muscle). The average Mn concentrations follow the trend of Fe, which correlates well with the similar function of those two metals [29]. Brain tissue exhibits higher amounts of Cu and Zn, while having rather low Fe content compared to other tissues. This fact underlines the role of Cu and Zn in neurological functions [30].

The concentrations found in the snap-frozen tissue samples are regarded to be most representative for the in vivo conditions. This is supported by the element concentrations, for example, found in lung tissue; the determined values correlate well with those reported earlier by Carvalho et al. [31] in a study using x-ray-based analysis techniques. Lopez-Alonso et al. [32] recently analyzed trace elements in commercial beef; liver and muscle tissue were examined in their study. Even though another species, the approximate trace metal concentrations should be comparable within the class of mammals. The found concentrations for transition metals correlate well with those found in the present study. Detected average concentrations found in heart tissue are in accordance with those reported earlier by Becker et al. in the same tissue type [33]. Similar findings were also reported for brain [5, 34]. All reported studies have used unfixed tissue. Thus, the obtained results are in good accordance with literature data and the comparison with the concentrations obtained for FFPE tissues should allow valid conclusions.

### Average trace element contents

**Fig. 2:**
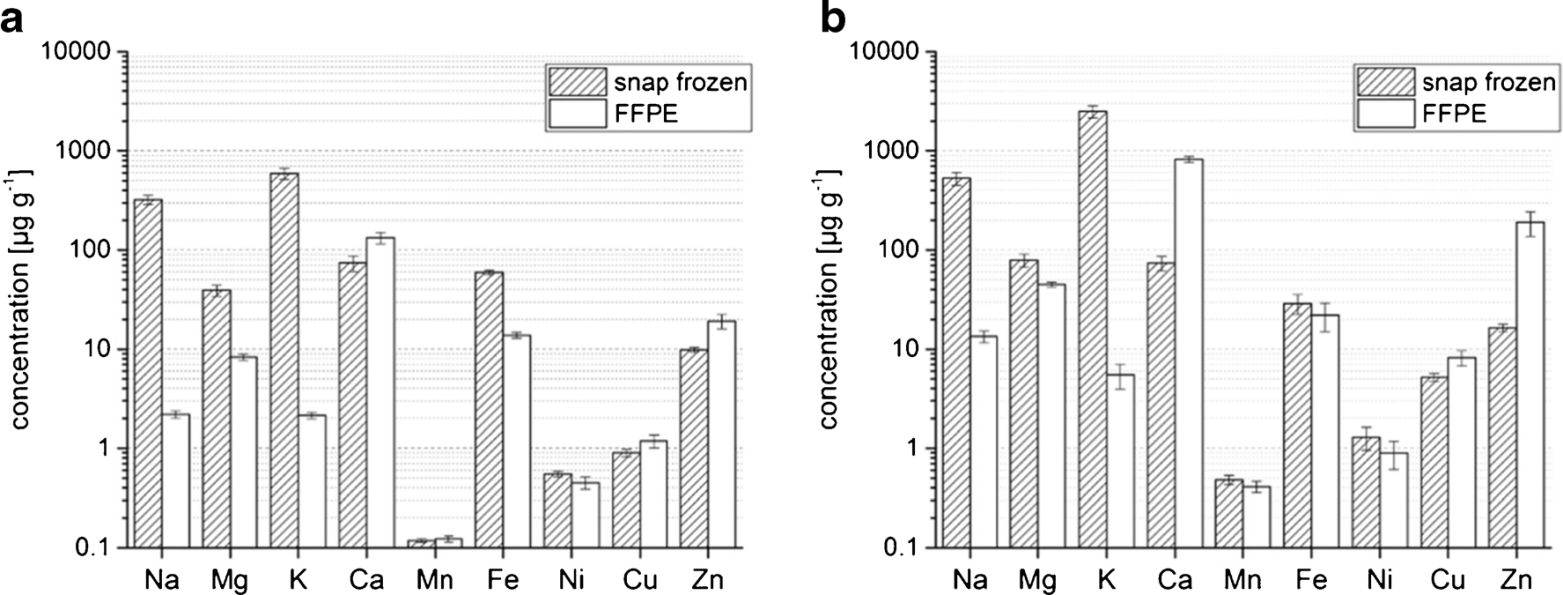
Comparison of the average metal concentrations between a snap-frozen and a FFPE sample of mouse lung (**a**) and mouse brain (**b**)

Due to the large number of sample preparation steps, the absolute element concentrations are expected to be influenced by the FFPE treatment. To demonstrate such possible wash-out effects in detail, the average concentrations derived from the elemental distribution images were considered. A full compilation of all values calculated from the LA-ICP-MS measurements is presented in Table 2. For better visualization, the data for lung and brain tissue were extracted to be represented in a graph, which is shown in Fig. 2, respectively. For better visualization, the concentrations are shown in logarithmic scale.

The largest difference between snap-frozen and FFPE samples can be found for the alkaline metal concentrations. With concentrations of some hundreds of micrograms per gram for Na and over 1000 μg g^−1^ for K, large amounts can be detected in the snap-frozen tissue samples. In comparison, values found in the FFPE samples are much lower. For K less than 1% recovery can be calculated in all investigated sample types; for Na, the recovery is in no case above 5%. This finding can be easily explained by the high mobility of alkaline metals in tissues: They occur completely in the form of free ions, unbound to larger molecular structures [28]. As a result, any aqueous solvents as used in parts of the FFPE process will readily wash these metals out from the tissue structure.

Less severe analyte loss could be found for Mg. Its concentration is generally lower in the FFPE samples, recoveries compared to the snap-frozen reference vary between 21% (lung) and 83% (muscle). This weaker wash-out compared to Na and K can be explained by looking at the speciation of Mg in biological systems: On average, about 30% of the total Mg occurs in free unbound form in a complete organism, while the rest is bound in complexes [35]; the exact ratio between free ionic and bound Mg depends on the tissue type. Similar to alkaline metals, free Mg can also easily be washed out from the tissue, whereas chelated Mg ions are more tightly bound to the macromolecular tissue matrix and are therefore less likely to be affected by the fixation and embedding process. A similar finding can be stated for iron. Average amounts found in snap-frozen brain, muscle, and liver tissue do not differ significantly from the respective FFPE samples. The other tissue types show significant analyte loss when comparing cryo-samples to FFPE. As for Mg, a portion of Fe is not bound to larger molecules [36]. This fraction can again be easily removed from the sample material by a solvent compared to the chelated fraction of Fe. The ratio between these two groups of species depends on the tissue type, explaining the different grades of wash-out.

The trace metals Mn, Ni, and Cu do not exhibit any significant concentration difference between the snap-frozen and the FFPE samples. Those metals are toxic to mammalian organisms when occurring in free, unbound form [37]. Thus, they appear in the organism almost completely bound to chelate complexes with different proteins or other macromolecules. High binding affinity makes it hard to break the chelate bonds by aqueous solvents. Thus, the concentration is barely affected by the FFPE sample preparation. The finding could be confirmed for all investigated tissue types.

In comparison to the metals having lower or similar concentration in the FFPE samples compared to the snap-frozen reference, Ca and Zn exhibit a higher average concentration in the FFPE samples of all investigated tissue types. Such increase is likely to be caused by contaminations introduced during any of the sample processing steps—another weakness of the FFPE sample preparation: Due to the vast number of process steps, a contamination of metals at the trace level can be easily introduced. Especially as the sample preparation is usually performed in clinical laboratories which might not be equipped according to the requirements for trace element investigations. Problems might be reagents with trace impurities as well as a workflow that introduces trace contaminations (e.g., the use of glassware). In the present case, the source of Ca and Zn could be easily identified. LA-ICP-MS measurements of thin cuts without having the embedding medium removed showed high Ca and Zn contents also in areas without tissue. Such Ca and Zn contamination originating from the embedding medium will also result in elevated metal contents in the sample, even after removing the paraffin.

### Comparison of element distributions

In order to evaluate if apart from the average contents also the lateral element distributions in different tissues are compromised by the FFPE process, tissue samples from one organ were compared. As indicated by the calculated average concentrations, FFPE sample preparation affects trace metals in the tissue. If bulk concentrations were altered, also some variations in element distributions were expected. In this part of the study, hematoxylin/eosin stain was used to identify relevant substructures in the respective tissues. Due to practical reasons, only selected tissue types and elemental distributions are shown.

**Fig. 3:**
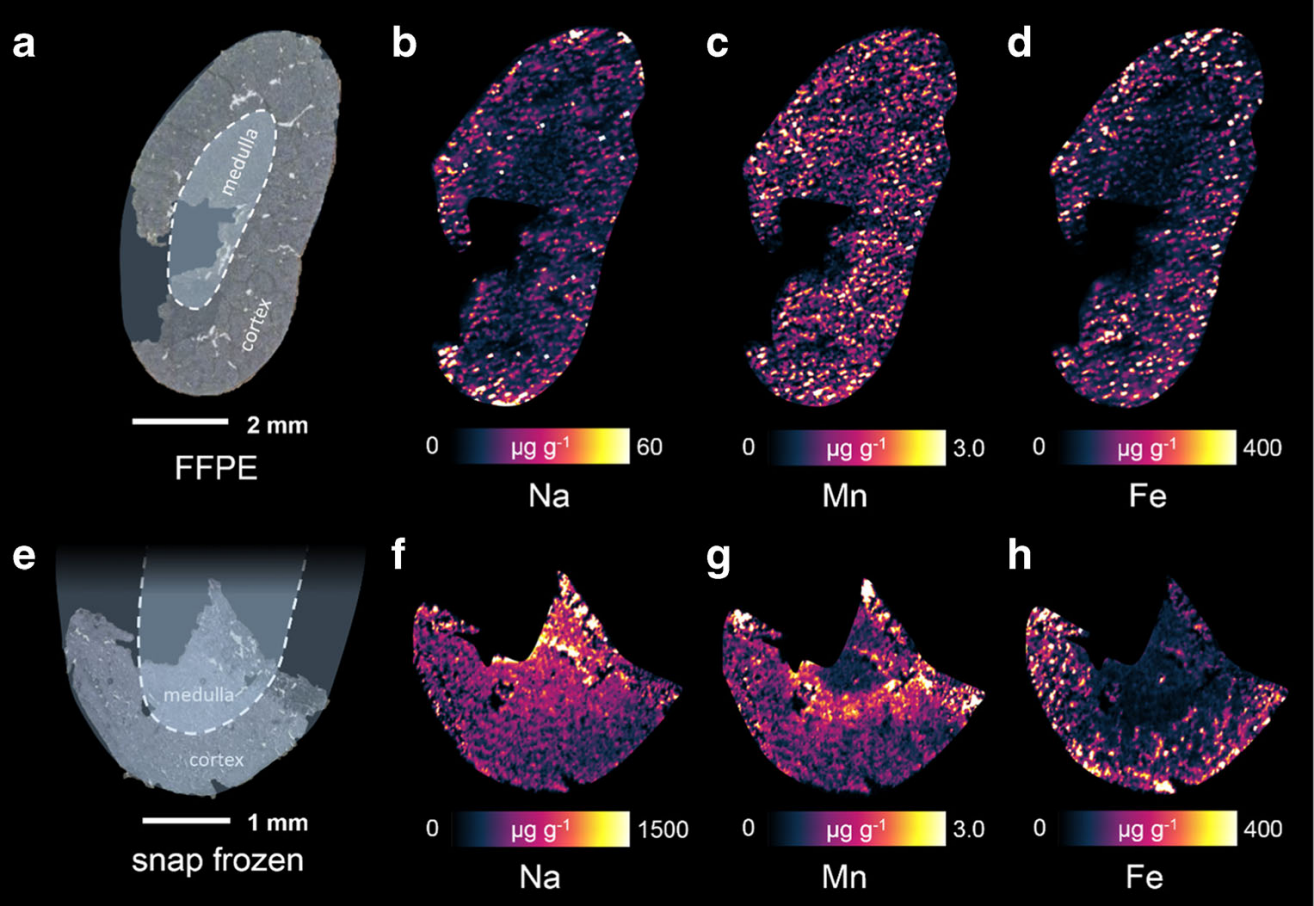
FFPE (**a**) and snap-frozen (**e**) samples of a mouse kidney with the elemental distributions of Na (**b**, **f**), Fe (**c**, **g**), and Mn (**d**, **h**)

A kidney sample is shown in Fig. 3. Microscopic scans with indication of the important substructures renal cortex and renal medulla are shown in Fig. 3a for the FFPE sample and in Fig. 3e for the corresponding snap-frozen sample from the very same kidney. It has to be pointed out that an incomplete section of the kidney is shown for the cryo-cut sample. This can be explained by difficulties which occurred during the sample preparation procedure where the organ had to be divided (cutting the organ correctly into two equal pieces). Additionally, correct positioning of the cryo-section on the sample carrier always poses a source for ruptures or other damages to the tissue. The position of the section relative to the complete kidney is indicated by the blue overlays. It has to be noted that the scale bars for Na are different, while the other elements have the same color scale for FFPE and cryo-cut sample. In general, the distribution of all three selected elements seems much more defined in the snap-frozen sample (Fig. 3f–h). The concentration of Na is slightly higher in the area of the medulla, whereas Fe and Mn are rather enriched towards the cortical structures. Similar distributions of Na and Mn were reported in another study [7]. Mn even shows a very distinct feature with a band of higher concentration near the renal pyramids, located at the corticomedullary junction. The increased concentration of Fe in the cortex can be explained by the higher density of blood vessels in contrast to the medulla. In comparison, the elemental distributions in the FFPE sample show a rather uniform distribution across the whole section, with Na and Fe showing slightly lower concentrations in the medulla. Even though less pronounced, the distributions of Fe in both sample preparation types are similar. The maximum concentration of Na decreases significantly from around 1500 to 60 μg g^−1^. Concentrations maxima of Fe and Mn in both samples are in a comparable range, supporting the finding from the investigations on the mean metal concentrations in the tissues. However, even if they are not removed from the tissue structure, also these elements seem to be influenced by the FFPE process, creating a slightly more blurred elemental distribution.

**Fig. 4:**
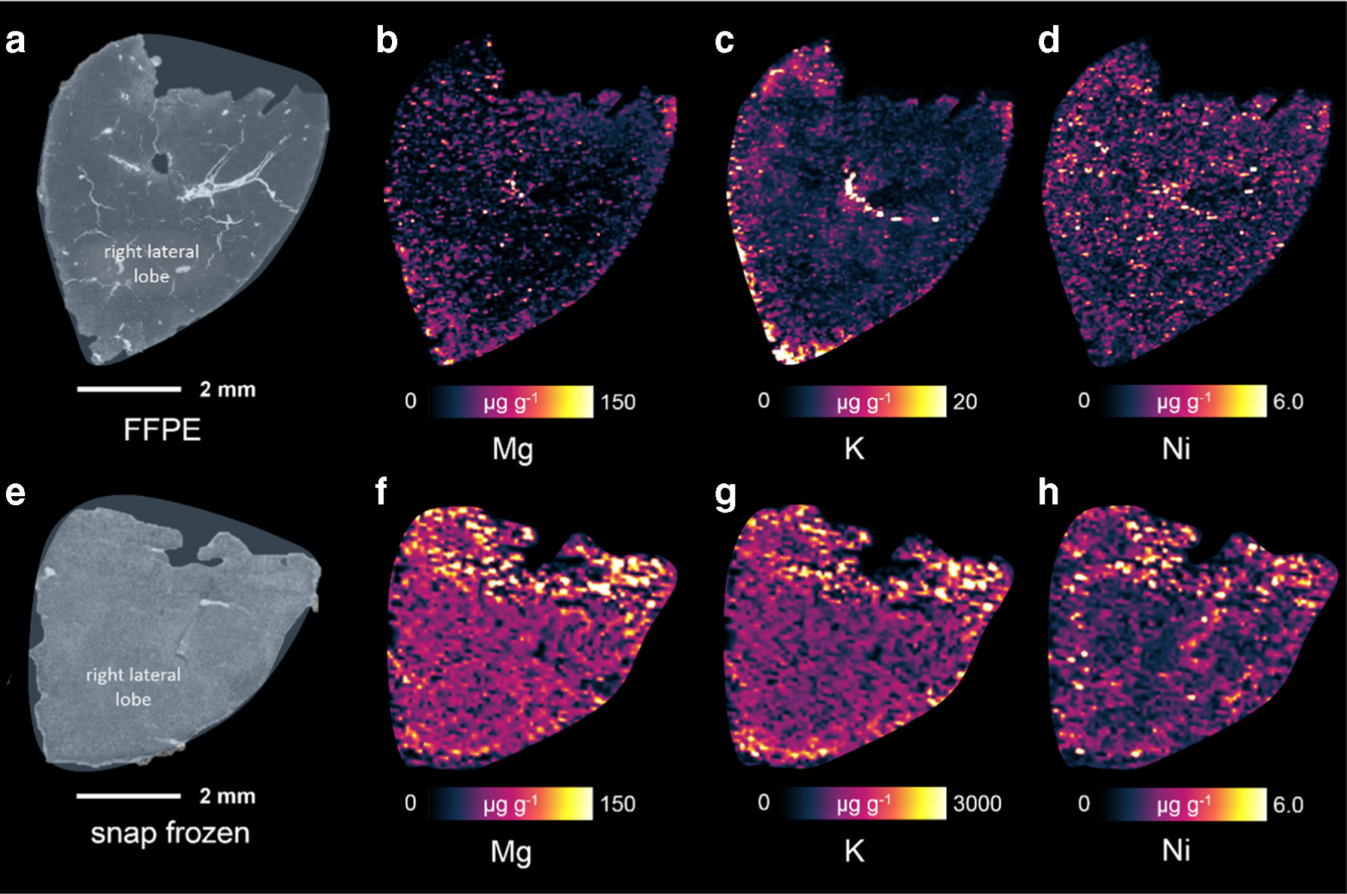
FFPE (**a**) and snap-frozen (**e**) samples of a mouse liver with the elemental distributions of Mg (**b**, **f**), K (**c**, **g**), and Ni (**d**, **h**)

A comparison of the trace element distributions in a snap-frozen and a FFPE liver sample is shown in Fig. 4. Both thin cuts represent the right lateral lobe of the liver, the similar shape can easily be recognized; no macroscopic substructures can be identified. Color scale bars for Mg and Ni are equally scaled for FFPE and cryo-cut samples, while K distribution images had to be scaled differently due to the large concentration differences between the sample preparation strategies. The elemental distributions are in general rather uniform in the snap-frozen section as well as in the FFPE sample. The distribution of Ni is slightly more structured in both samples; similar distribution could be found, for example, for Fe. This corresponds well with findings reported earlier for trace elements in the liver [4]. One semicircular area of higher potassium concentration in the center of the FFPE sample indicates the position of a large blood vessel. This feature is also represented by other metal distributions such as Mn and Cu (not shown). However, it does not appear in the snap-frozen sample, which makes a direct comparison of the elemental distributions in this specific area impossible. Besides that, the potassium distribution in the FFPE sample also shows high concentrations in the outer sample areas. This appearance is not represented in the snap-frozen sample. Therefore, it is very likely that the distribution obtained from the FFPE sample is influenced by the pre-treatment of the sample, leading to wash-out of analytes. Samples with the remaining higher concentrations might have higher density or hydrophobicity which hampers the release of analyte into the aqueous solvent. These findings were observed for all replicate sample measurements.

All other investigated organs show similar results: Elemental distributions are usually more homogeneous in the FFPE samples, a fact that can be well explained by diffusional processes occurring during fixation and embedding. However, the analyte distortion is less pronounced for transition metals than for alkaline elements; the strength of the effect of the embedding process on Mg lies between those two groups. Results of this comparative distribution study indicate that FFPE samples can be used only to a limited extent for elemental distribution studies. The qualitative distributions of many elements seem to be biased towards more homogeneous distributions compared to the snap-frozen samples, which are believed to represent the actual in vivo conditions to a good extent.

## Summary

FFPE is a widely used preparation strategy for tissue specimen. Due to the large availability of samples in archives, it would be interesting if these samples could also be used for elemental distribution studies. In the comparison with snap-frozen tissues which were used as reference samples, elemental distributions as well as average metal concentrations showed to be partly altered in the FFPE samples, which restricts the general applicability of FFPE sample preparation in metallomics studies. FFPE samples showed to be completely unsuitable, if the investigation of alkaline metals is required; distributions as well as absolute amounts were heavily influenced. Especially the relative distributions of some transition metals as well as their bulk concentrations showed to be altered to a lesser extent in the FFPE samples. These results indicate that if only the analysis of transition metals is required, also FFPE samples can be used; even if finer structures appeared to be blurred, the general elemental distributions were comparable. For Ca and Zn, contaminations introduced during the embedding process could be identified, highlighting that metal analysis in FFPE samples still has to be carefully considered in order not to obtain results which are biased by the sample preparation process. However, even if snap-frozen samples without fixation definitely provide the most accurate way for analysis of metals in tissue samples, FFPE samples can also deliver valid quantitative elemental distribution results.
